# Evaluation of the Expression of Infection-Related Long Noncoding RNAs among COVID-19 Patients: A Case-Control Study

**DOI:** 10.1155/2024/3391054

**Published:** 2024-02-15

**Authors:** Zahra Firoozi, Elham Mohammadisoleimani, Farzaneh Bagheri, Atefeh Taheri, Babak Pezeshki, Mohammad Mehdi Naghizadeh, Abdolreza Daraei, Jalal Karimi, Yousef Gholampour, Yaser Mansoori, Zahra Montaseri

**Affiliations:** ^1^Department of Medical Genetics, Fasa University of Medical Sciences, Fasa, Iran; ^2^USERN Office, Fasa University of Medical Sciences, Fasa, Iran; ^3^Department of Medical Biotechnology, Fasa University of Medical Sciences, Fasa, Iran; ^4^Department of Microbiology and Virology, School of Medicine, Kerman University of Medical Sciences, Kerman, Iran; ^5^Department of Internal Medicine, Fasa University of Medical Sciences, Fasa, Iran; ^6^Department of Tissue Engineering and Regenerative Medicine, Faculty of Advanced Technologies in Medicine, Iran University of Medical Sciences, Tehran, Iran; ^7^Noncommunicable Diseases Research Center, Fasa University of Medical Sciences, Fasa, Iran; ^8^Department of Medical Genetics, School of Medicine, Babol University of Medical Sciences, Babol, Iran; ^9^Department of Infectious Diseases, School of Medicine, Fasa University of Medical Sciences, Fasa, Iran

## Abstract

**Background and Aims:**

Severe acute respiratory syndrome coronavirus 2 (SARS-CoV-2), a worldwide pandemic, activates signaling cascades and leads to innate immune responses and secretion of multiple chemokines and cytokines. Long noncoding RNAs (lncRNAs) have a crucial role in inflammatory pathways. Through our search on the PubMed database, we discovered that existing research has primarily focused on examining the regulatory impacts of five lncRNAs in the context of viral infections. However, their role in regulating other conditions, including SARS-CoV-2, has not been explored. Therefore, this study aimed to investigate the expression pattern of lncRNAs in the peripheral blood mononuclear cells (PBMC) and their potential roles in SARS-CoV-2 infection. Potentially significant competing endogenous RNA (ceRNA) networks of these five lncRNAs were found using online in-silico techniques.

**Methods:**

Ethylenediaminetetraacetic acid (EDTA) blood samples of the control group consisted of 45 healthy people, and a total of 53 COVID-19-infected patients in case group, with a written informed consent, was collected. PBMCs were extracted, and then, the RNA extraction and complementary DNA (cDNA) synthesis was performed. The expression of five lncRNAs (lnc ISR, lnc ATV, lnc PAAN, lnc SG20, and lnc HEAL) was assessed by real-time PCR. In order to evaluate the biomarker roles of genes, receiver operating characteristic (ROC) curve was drawn.

**Results:**

Twenty-four (53.3%) and 29 (54.7%) of healthy and COVID-19-infected participants were male, respectively. The most prevalent symptoms were as follows: cough, general weakness, contusion, headache, and sore throat. The results showed that three lncRNAs, including lnc ISR, lnc ATV, and lnc HEAL, were expressed dramatically higher in the case group compared to healthy controls. According to ROC curve analysis, lnc ATV has a higher AUC and is a better biomarker to differentiate COVID-19 patients from the healthy controls. Then, using bioinformatics methods, the ceRNA network of these lncRNAs enabled the identification of mRNAs and miRNAs with crucial functions in COVID-19.

**Conclusion:**

The considerable higher expression of ISR, ATV, and HEAL lncRNAs and the significant area under curve (AUC) in ROC curve demonstrate that these RNAs probably have a potential role in controlling the host innate immune responses and regulate the viral replication of SARS-CoV-2. However, these assumptions need further in vitro and in vivo investigations to be confirmed.

## 1. Introduction

SARS-CoV-2 was first discovered in Wuhan, China, in December 2019 and rapidly became a pandemic all around the world [1–3]. COVID-19 clinical manifestations vary widely among patients from asymptomatic to high severe states [4]. The most frequent signs and symptoms of COVID-19 are fever, shortness of breath, nonproductive cough, sore throat, and diarrhea [5].

Following the first exposure of SARS-CoV-2 to the host immune system, several receptors including toll-like receptors (TLRs) and NOD-like receptors (NLRs), retinoic acid-inducible gene-I (RIG-I)-like receptors (RLRs), and cyclic GMP-AMP synthase (cGAS) detect the viral particles. Afterwards, signaling cascades result in activation of innate immune responses and secretion of multiple chemokines and cytokines [6–8]. Some studies have shown increased levels of different cytokines and chemokines such as tumor necrosis factor a (TNF-*α*), interleukin 6 (IL-6), interleukin 7 (IL-7), C-X-C motif chemokine ligand 10 (CXCL10), C-C motif chemokine ligand 2 (CCL2), C-C motif chemokine ligand 3 (CCL3), and *α*-chain of IL-2 receptor [9, 10]. Nod-like receptor pyrin domain-containing 3 (NLRP3) is a major inflammasome which is involved in cytokine storm and intensive clinical manifestations of COVID-19 [11].

Almost 80% of eucaryotic genome is composed of noncoding sequences that are consequently transcribed to noncoding RNAs (ncRNAs) [12]. These RNAs are categorized into two groups as follows: short ncRNAs with a length <200 nucleotides and long noncoding RNAs (lncRNAs) with a length over 200 nucleotides [13]. Although most of the lncRNAs are not protein-coding, they are involved in modulation of transcription patterns and regulation of different protein activities [13–16]. LncRNAs have a crucial role in inflammatory pathways and inflammasomes [17]. LncRNAs are involved in cellular and molecular procedures and have shown to act as epigenetic regulators in several key molecular processes [18]. By exposing a sponge effect on miRNAs, lncRNAs can control the mRNAs that target them [19]. Recently, it has been showed that interactions of noncoding RNAs (ncRNAs), such as microRNAs (miRNAs), and lncRNAs are related to SARS-CoV-2 infection, so, in this research, we have also focused on the interactions of ncRNAs.

Angiotensin-converting enzyme (ACE) which is the receptor of SARS-CoV-2 is highly expressed in lungs, testis, and kidneys [20]. The lncRNAs and miRNAs are able to affect ACE2 expression [21]. ACE2 is expressed in humans and is also altered in many viral infections, such as the hepatitis C virus (HCV), the hepatitis B virus (HBV), the influenza virus, the human immunodeficiency virus (HIV), the herpes simplex virus (HSV), and the SARS-CoV-2 [22–24].

We scanned the PubMed database for a list of recently discovered lncRNAs that have been validated by other research. Thus, interferon-stimulated lncRNA (lncRNA ISR), lncRNA ATV, PA-associated noncoding RNA (lncRNA PAAN), interferon-stimulated gene 20 (lncRNA ISG20), and HIV-1-enhanced lncRNA (lncRNA HEAL) were the five lncRNAs that were chosen. We found that while current research has concentrated on the regulatory effects of five lncRNAs in viral infection, their involvement in regulating other disorders, such as SARS-CoV-2, has not been investigated by searching the PubMed database.

We searched for ceRNA networks on which in-silico studies had been performed in viral infections. The roles of lnc ISR and lnc PAAN have been proven with bioinformatics studies [25]. For example, lnc ISR is a lncRNA that is expressed concurrently with influenza A virus (IAV) infection as a result of interferon (IFN) signaling [26, 27]. IFN signaling is implicated in both the protective and damaging aspects of SARS-CoV-2 [28]. According to the findings of Wang et al., lnc PAAN facilitates IAV replication [29]. In line with a study by Chai et al., the expression of lnc ISG20 is suppressed by IFN-*β* stimulation, which inhibits the replication of IAV [30]. Lnc ATV is another lncRNA that is substantially expressed when IFN types I and III are present. It is also elevated in viral infections such as Zika and hepatitis C [31]. Additionally, throughout the active phase of HIV infection, the expression of lnc HEAL increases in T-cell lymphocytes, microglia, and monocyte-derived macrophages [32]. Some studies have been performed on the status of lncRNAs during COVID-19 infection. Previous studies showed that the expression of some lncRNAs is dysregulated in SARS-CoV-2-infected cell lines and these RNAs might be involved in immune response against COVID-19 [25, 33, 34]. Thus, this study aimed to investigate the potential roles of lncRNAs in SARS-CoV-2 infection and the lncRNA profiles among COVID-19 patients in comparison to the healthy control group.

## 2. Material and Methods

### 2.1. Sample Collection

A case-control study was performed from January to March 2021. There were 45 healthy people in the control group and 53 COVID-19-infected patients in the case group. The healthy group consists of individuals who had no infectious or immune-related diseases, such as autoimmunity, allergy, cancer, and liver diseases, and these subjects are negative in RT-PCR test results. RT-PCR, the gold-standard method for COVID-19 diagnosis, was also performed to detect SARS-CoV-2 infection. This study was performed in Valiasr Hospital, Fasa, Fars Province. EDTA blood samples and written informed consent were collected from all participants. The ethical approval was obtained from the ethic committee of Fasa University of Medical Sciences (IR.FUMS.RES.1399.040).

### 2.2. RNA Extraction and cDNA Synthesis

PBMC extraction was performed using Lymphodex, Inno-Train, Germany extraction kit. The RNA was extracted using RiboEx, GeneAll (Cat. No. 301-902). After RNA extraction, cDNA was synthesized by BioFact cDNA synthesis kit (Cat. No. BR441-096).

### 2.3. Real-Time PCR

The expression of noncoding RNAs was evaluated by SYBR® Green real-time PCR. The internal control for quantitative applications of lncRNAs was actin beta (ACTB). The noncoding RNAs and housekeeping gene (ACTB) primers are listed in Table 1. Real-time PCR was performed using Ampliqon master mix (Cat. No. A325402-25) in total volume of 15 *μ*l, as follows: 7.5 *μ*l master mix, 1 *μ*l of cDNA, 0.75 *μ*l of each primer, 5 *μ*l DNase-free dH2O for 45 cycles of 95°C for 20 seconds, and then 60°C for 30 seconds.

### 2.4. Data Analysis

The expression of each noncoding RNA was interpreted based on the 2^−ΔΔCT^ (fold change) method. Before using this method, amplification efficiency was assessed by standard curve. The statistical data were analyzed using SPSS V.22 software. The nonparametric Mann–Whitney test was used to compare the expression between the normal control group and the group infected with COVID-19. We used the pROC package in R software to draw the ROC curves for differentially expressed genes and calculate the AUC. Larger AUC value means the gene can well distinguish COVID-19 patients from the healthy controls, and this gene is a better biomarker. Comparison of groups in terms of age and gender was carried out with Chi-square statistical test. *P* value less than 0.05 was considered significant.

### 2.5. In-Silico Study

Interactions between lncRNAs and miRNAs were predicted using the DIANA-LncBase v3 (https://diana.e-ce.uth.gr/lncbasev3) database, while interactions between miRNAs and mRNAs were predicted using TargetScan (https://www.targetscan.org/vert_80/) and mirTarBase (https://mirtarbase.cuhk.edu.cn/~miRTarBase/miRTarBase_2022/php/index.php) databases. By using this method, we were able to identify lncRNA-miRNA and miRNA-mRNA pairings. Furthermore, the STRING database (https://string-db.org) was utilized to investigate PPI (protein-protein interaction). The Cytoscape (version 3.10.1) was used to view the data.

## 3. Results

### 3.1. Demographic Data

24 (53.3%) and 29 (54.7%) of healthy and COVID-19-infected participants were male, respectively. The mean age of the healthy group was 50.5 ± 20.9, while it was 44.0 ± 16.0 for patients' group. Almost half (47.2%) of patients and 31.1% of healthy people were over 50 years old.

### 3.2. Clinical Signs and Symptoms

COVID-19 patients showed variable clinical manifestations. The most prevalent symptoms were as follows: cough, general weakness, contusion, headache, and sore throat, which were observed in over 50% of patients. On the other hand, nausea, vomiting, diarrhea, conjunctivitis, and stomachache were rarely reported. Six (11.6%) of patients had underlying diseases (Table 2).

### 3.3. Expression Pattern of lncRNAs

**Figure 1:**
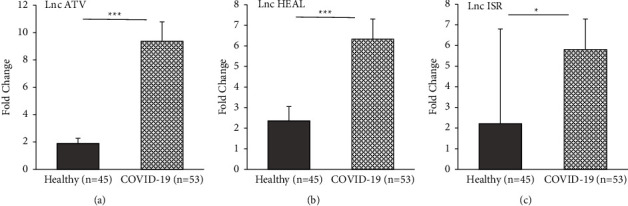
Box and whisker plot of expression of lncRNAs in COVID-19-infected patients and control group. (a) lncRNA ATV, (b) lncRNA HEAL, and (c) lncRNA ISR. The grey columns show the expression of lncRNAs in healthy participants, and the dotted columns show the expression of lncRNAs in COVID-19-infected patients (^*∗*^*P* ≤ 0.05; ^*∗∗∗*^*P* ≤ 0.001).

**Figure 2:**
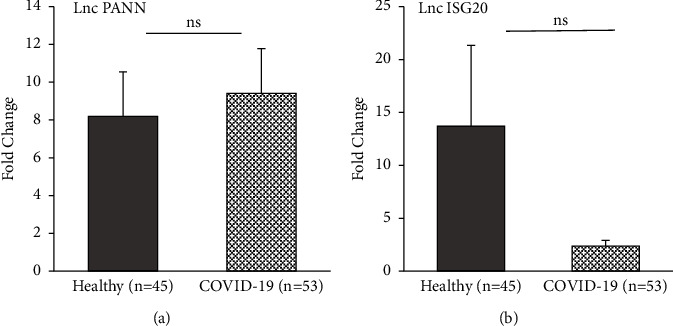
Box and whisker plot of expression of lncRNAs in COVID-19-infected patients and control group. (a) lncRNA PAAN and (b) lncRNA ISG20. The grey columns show the expression of lncRNAs in healthy participants, and the dotted columns show the expression of lncRNAs in COVID-19-infected patients (ns: *P* > 0.05).

Real-time PCR was used to evaluate the expression of five noncoding RNAs including lnc ISR, lnc ATV, lnc PAAN, lnc SG20, and lnc HEAL. According to the findings, the expression levels of several lncRNAs were significantly different in COVID-19-infected patients compared to healthy controls. Two lncRNAs, including lncRNA ATV (*P* ≤ 0.001) and lncRNA HEAL (*P* ≤ 0.001), were expressed dramatically higher in the case group compared to healthy controls (Figures 1(a) and 1(b)). Moreover, the expression of lncRNA ISR was significantly higher in COVID-19-infected patients (*P* ≤ 0.05), see Figure 1(c). No significant different expression of lncRNA PAAN and lncRNA ISG20 was observed between two groups (*P* > 0.05) (Figures 2(a) and 2(b)). The detailed statistical analyses are shown in Table 3.

**Figure 3:**
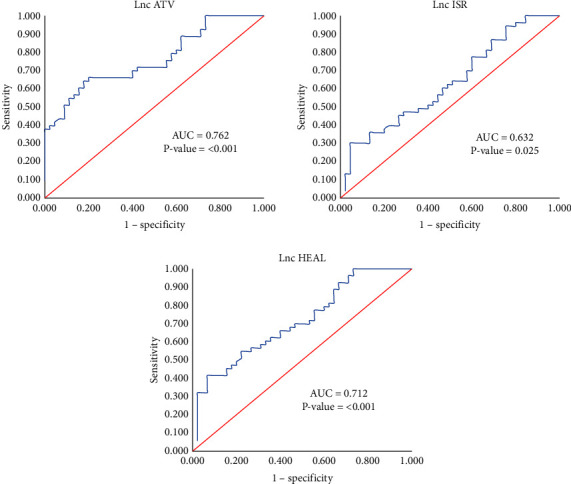
ROC curve analysis. Lnc ATV has a higher AUC (AUC = 0.762) and is a better biomarker to differentiate COVID-19 patients from the healthy controls.

According to our ROC curve analysis, lnc ATV has a higher AUC (AUC = 0.762) which represented it might be a great biomarker to differentiate COVID-19 patients from the healthy controls (Figure 3).

### 3.4. CeRNA Network

**Figure 4:**
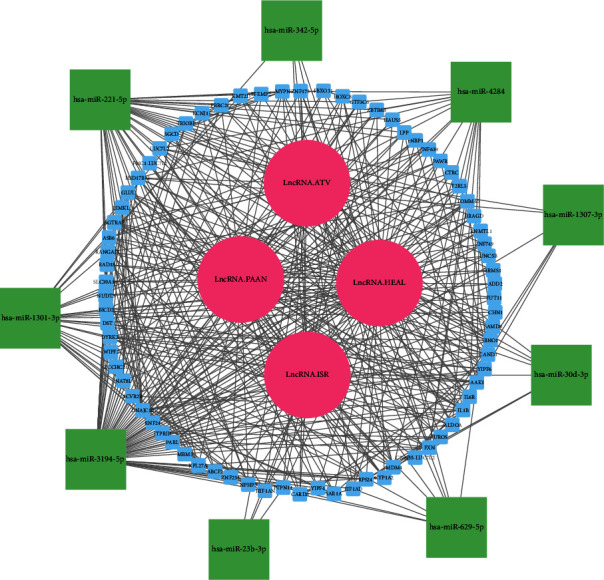
Based on lncRNA-miRNA pairings, miRNA-mRNA pairs, and PPI, the lncRNA-miRNA-mRNA triple regulatory network was constructed. mRNAs are shown as blue, miRNAs as green, and lncRNAs as pink.

The lncRNAs/miRNAs/mRNAs regulatory network was constructed by in-silico investigation. The network was constructed based on 4 lncRNAs (in the online database, lnc SG20 did not interact with any miRNA), 15 miRNAs, and 207 mRNAs (Figure 4). We identified 15 lncRNA/miRNA interaction pairs, 167 miRNA/mRNA interaction pairs, and 270 protein-protein interaction (PPI) pairs. The RNF24, F2RL3, and ACVR2B, and hsa-miR-23b-3p, hsa-miR-629-5p, hsa-miR-30d-3p, hsa-miR-1307-3p, hsa-miR-342-5p, and hsa-miR-221-5p had the highest level of interaction between the mRNAs and miRNAs in this network, respectively.

## 4. Discussion

One of the most contagious viruses, SARS-CoV-2, is a member of the Coronaviridae (CoV) family, which has become a pandemic and is linked to high rates of morbidity and mortality [35]. The increasing amount of evidence suggests that the host transcriptome changes following the viral infections and several signaling pathways are stimulated. For instance, Merkel cell carcinoma (MCC) is caused by Merkel cell polyomavirus (MCPyV), which is a small DNA tumor virus and oncogenic virus [36]. In recent years, a long type of noncoding RNAs known as lncRNAs has attracted incredible attention. LncRNAs are one of the major regulators of antiviral immune responses [26].

Numerous lncRNAs have been shown to be differently expressed in COVID-19 patients, and important lncRNAs for virus-host interactions have also been identified, along with improvements in research instruments and methodologies. Furthermore, targeting gene transcription and protein translation are the main targeted therapies for SARS-CoV-2-infected cells, based on the properties of poorly conserved lncRNAs that are extensively involved in cell proliferation, differentiation, apoptosis, and other biological processes. Moreover, a number of bioanalysis-based investigations have discovered a variety of dysregulated lncRNAs connected to SARS-CoV-2 replication [33]. According to some research, the pathogenesis of SARS-CoV-2 is facilitated by epigenetic changes in both individual genomes and the virus. Nongenomic alterations in gene expression and function are heritable known as epigenetic modifications. Deoxyribonucleic acid (DNA) methylation, histone changes, and noncoding RNA-associated gene silencing are three key epigenetic processes [37].

The lncRNAs listed below are examples of those with the mentioned previously functions. In a study, Di Qu et al. introduced the lncRNA GM26917 in HIV-1 by sponging the miR-124-3p. The lncRNA NORAD is implicated in cytokine storms because it has the ability to target five of the ten cytokines that are engaged in them. Additionally, new research on HIV-1 has shown that MALAT1 regulates promoter-enhancer interactions to enhance viral transcription and infection [38]. It is interesting to note that lncRNAs can also control the SARS-CoV-2 innate immune response by connecting to the IFN pathway. IFN-1 response was shown to be considerably downregulated in the ncRNA regulatory network in a recent study employing total transcriptome RNA sequencing in COVID-19 patients [39]. In the context of viral infections, particularly COVID-19, the regulation and function of lncRNAs are intricate and can be changed based on a number of variables, including the infection's stage, the viral strain, and individual characteristics. In line with our analysis, previous research has indicated that upregulated lncRNA-NEAT1 and lncRNA-TUG1 could influence cytokine storms in both moderate and severe forms of COVID-19 infection [40].

IFNs are a family of secreted proteins that are able to hinder viral infection and replication. Janus kinase-signal transducer and activator of transcription (Jak-STAT) signaling cascade leads to transcription of several IFN-stimulated genes. In the context of SARS-CoV-2, IFN signaling is involved in both protective and detrimental aspects of this infection [41]. Thus, assessment of IFN-associated genes might provide a new insight into the pathogenesis of SARS-CoV-2. The majority of studied lncRNAs in the present study were IFN-stimulated genes including lnc ISR, lnc ATV, and lnc ISG20 [27, 30, 31].

Lnc ISR is a lncRNA which is synergically expressed along with IAV infection, by IFN signaling. On the other hand, hosts with type I IFN receptor (IFNAR1) deficiency, lnc ISR is not induced. It has been observed that silencing of lnc ISR results in replication of IAV. Thus, lnc ISR and IFN signaling are involved in antiviral immune responses. In other words, lnc ISR suppresses the growth and replication of IAV [26, 27]. Next, expression of lncRNA ISR was assessed in mouse tissues and cell lines infected with or without IAV, and their results showed that lncRNA ISR expression was significantly increased after the IAV infection [27]. Our experimental results indicate that lnc ISR is upregulated in COVID-19 patients compared to another group, similar to the study of Qidong Pan et al. Also, this lncRNA with significant AUC in ROC curve could have a biomarker role.

Lnc ATV is another lncRNA that is highly expressed under the influence of IFN type I and III. In a study by Jingjing Fan et al., microarray was used to determine the changes in host lncRNA expression in Huh7 cells stimulated by type I or type III IFNs, and their results demonstrated that 272 lncRNAs were upregulated while 631 were downregulated. In the next step, lnc ATV was selected and its expression was assessed by qRT-PCR. IFN*α*2b and IFN*λ*1 significantly upregulated the expression of endogenous lnc ATV in Huh7 cells. Furthermore, silencing of this lncRNA inhibits the viral replication in several RNA viruses such as Zika virus, hepatitis C virus, Sendai virus, and Newcastle disease virus. Reciprocally, the knockdown of lnc ATV leads to enhanced activity of IFN and RIG-I antiviral signaling pathways. Thus, this human-specific lncRNA has a considerable role in suppression of host innate immunity during viral infection [31]. In our study, this lncRNA was also significantly expressed higher in COVID-19 patients, like Huh7 cells. Moreover, it is stated that the IFN I/III response changes in animal models of COVID-19- and SARS-CoV-1-infected patients [25]. Hence, this study confirms our observation regarding the higher expression of lnc ATV in COVID-19 patients that might be resulted from IFN I/III response.

The nomenclature of lnc HEAL refers to its role in HIV-1 infection. The expression of lnc HEAL increases in T cell lymphocytes, microglia, and monocyte-derived macrophages during active phase of HIV infection, while its expression is downregulated in HIV-infected latent CD4+T lymphocytes [32]. The complex of lnc HEAL and FUS RNA-binding protein accelerates HIV replication. It has been reported that knockdown of lnc HEAL or disturbing the HEAL-FUS complex can be employed as a cure for AIDS and helps eradication of HIV reservoir, but the strategies are still unidentified [42]. Our experimental results indicate that lnc HEAL is upregulated in COVID-19 patients compared to the control group, similar to its expression in different cells during active phase of HIV infection. Also, this lncRNA with significant AUC in ROC curve could have a biomarker role.

Although no significant relation was observed between the expression of lnc PAAN and lnc ISG20 and SARS-CoV-2 infection, Wang J et al. reported that lnc PAAN enhances the replication of IAV. But similar to our results, this lncRNA was not altered during infection with enterovirus 71, VSV-Gpseudo-type of HIV-1, and Zika virus [29]. It seems that lnc PAAN is a specific prognostic marker for IAV infection.

Lnc ISG20 hinders the replication of HBV via exonuclease activity through binding to the epsilon stem loop of HBV RNA. In concordance with these findings, the IFN-gamma (IFN-*γ*) is used for treatment of HBV by blocking the viral replication [41]. On the other hand, a study conducted by Chai et al. revealed that silencing of lnc ISG20 leads to higher viral replication. Indeed, lnc ISG20 is a negative regulator of IAV replication [30]. Although no significant association was found between the expression of lnc ISG20 and SARS-CoV-2, the expression of this lncRNA was lower in COVID-19 patients in comparison to healthy patients, which seems that there might be a reverse association between lnc ISG20 and replication of SARS-CoV-2.

Furthermore, in order to better understand the significance of these lncRNAs in COVID-19 progression, we designed the ceRNA regulatory network of these lncRNAs using online databases. Also, bioinformatics construction of the lncRNA/miRNA/mRNA network suggests that the RNF24, F2RL3, ACVR2B, and hsa-miR-23b-3p, hsa-miR-629-5p, hsa-miR-30d-3p, hsa-miR-1307-3p, hsa-miR-342-5p, and hsa-miR-221-5p had the most interaction among the mRNAs and miRNA, respectively [43–50]. Furthermore, a few of these genes have significant involvement in the development and prognosis of COVID-19, and their interactions with lncRNAs highlight their significance. LncRNAs have a wide range of roles in pathogenesis, and current research on COVID-19 has focused on interferons and cytokine storms [34]. With the aid of the potential gene network created by bioinformatics, these investigations may serve as a precursor to future discoveries about the functions of these noncoding genes in COVID-19. According to our knowledge, this is the first investigation to develop a novel perspective on the functions of these lncRNAs in regulating the immune response to SARS-CoV-2. Nonetheless, additional research is necessary to investigate the clinical ramifications of these findings.

## 5. Conclusion

The considerable higher expression of ISR, ATV, and HEAL lncRNAs demonstrates that these RNAs probably have a remarkable role in the host innate immune responses and the viral replication of SARS-CoV-2. These three lncRNAs need to be knocked down to observe their effect on innate immune response and signaling pathways. Hence, these assumptions need further in vitro and in vivo investigations to be confirmed.

## Figures and Tables

**Table 1:** List of primers used in this study.

Gene names	Sequences	Amplicon size
ATV-F	AGGAGCAAGATTCCAACATCA	117
ATV-R	TCCAATCTGATTCCTCGTTTCT	

PAAN-F	GGGCATCCAGTTCCAATCCA	173
PAAN-R	AGAAACACGGTGGACACCTC	

ISG20-F	GCATCCCGACATTGGTTTA	75
ISG20-R	AGCTGCAGGATCTACTTACAGAC	

ISR-F	ATGCATCCCTGCAAACCCAT	85
ISR-R	GGGACTGCTGGTGTAAGACG	

HEAL-F	GTATCTCACCGTCCCAGAATG	120
HEAL-R	GAGATGAACCCTCTGCTTGTC	

ACTB-F	TGGAACGGTGAAGGTGACAG	129
ACTB-R	CTGTAACAACGCATCTCATATTTGG	

**Table 2:** Clinical signs and symptoms of COVID-19-infected patients.

Clinical symptoms	No *n* (%)	Yes *n* (%)
Fever and chills	30 (56.6%)	23 (43.4%)
Cough	19 (35.8%)	34 (64.2%)
Shortness of breath	36 (67.9%)	17 (32.1%)
General weakness	19 (35.8%)	34 (64.2%)
Contusion	22 (41.5%)	31 (58.5%)
Confusion or Irritability	38 (71.7%)	15 (28.3%)
Sore throat	26 (49.1%)	27 (50.9%)
Runny nose	38 (71.7%)	15 (28.3%)
Diarrhea	50 (94.3%)	3 (5.7%)
Nausea and vomiting	48 (90.6%)	5 (9.4%)
Headache	24 (45.3%)	29 (54.7%)
Chest pain	46 (86.8%)	7 (13.2%)
Stomach ache	47 (88.7%)	6 (11.3%)
Joints' pain	39 (73.6%)	14 (26.4%)
Conjunctivitis	50 (94.3%)	3 (5.7%)
Fatigue	43 (81.1%)	10 (18.9%)
Comorbidities	47 (88.7%)	6 (11.3%)

**Table 3:** Comparison of lncRNAs expression between healthy people and COVID-19 patients.

Noncoding RNAs	Fold change						
	Healthy			COVID-19			*P* value Mann–Whitney
	Mean	SD	Median	Mean	SD	Median	
LncRNA ISR	2.21	4.80	0.90	4.20	5.80	1.44	**0.025**
LncRNA ATV	1.89	2.55	1.25	9.37	10.33	5.32	**<0.001**
LncRNA PAAN	8.20	15.75	1.29	9.41	17.29	1.89	0.139
LncRNA ISG20	13.71	51.26	0.70	2.36	4.04	0.30	0.379
LncRNA HEAL	2.36	4.66	1.14	6.33	7.11	3.15	**<0.001**

Bold numbers show the significant parameter.

## Data Availability

The data supporting the findings of this study are included within the article.
